# Anorexia and bulimia in relation to ulcerative colitis: a Mendelian randomization study

**DOI:** 10.3389/fnut.2024.1400713

**Published:** 2024-07-10

**Authors:** Qiang Su, Jian Li, Yun Lu, Min Wu, Jiang Liang, Zhenxiang An

**Affiliations:** ^1^The First Clinical Medical College of Guizhou University of Traditional Chinese Medicine, Guiyang, China; ^2^The First Affiliated Hospital of Guizhou University of Traditional Chinese Medicine, Guiyang, China

**Keywords:** eating disorder, anorexia, bulimia, ulcerative colitis, Mendelian randomization

## Abstract

**Background:**

Evidence for anorexia and bulimia in relation to the risk of ulcerative colitis (UC) is limited and inconsistent. The objective of this research was to utilize bi-directional, two-sample Mendelian randomization (MR) analysis to predict the causal association between anorexia nervosa and bulimia nervosa with UC.

**Methods:**

The genome-wide association studies (GWAS) provided data for anorexia and bulimia from the UK Biobank, utilizing single-nucleotide polymorphisms (SNP) as instrumental variables. Additionally, genetic associations with UC were collected from various sources including the FinnGen Biobank, the UK Biobank and the International Inflammatory Bowel Disease Genetics Consortium (IIBDGC). The main analytical approach utilized in this study was the inverse-variance-weighted (IVW) method. To evaluate horizontal pleiotropy, the researchers conducted MR-Egger regression and MR-PRESSO global test analyses. Additionally, heterogeneity was assessed using the Cochran’s *Q* test.

**Results:**

This study found a negative association between genetically predicted bulimia (OR = 0.943, 95% CI: 0.893–0.996; *p* = 0.034) and the risk of UC in the IIBDGC dataset, indicating that individuals with bulimia have approximately a 5.7% lower risk of developing UC. No association was observed in the other two datasets. Conversely, genetically predicted anorexia was not found to be causally associated with UC. In bi-directional Mendelian randomization, UC from the IIBDGC dataset was negatively associated with the risk of anorexia (OR = 0.877, 95% CI: 0.797–0.965; *p* = 0.007), suggesting that UC patients have approximately a 12.3% lower risk of developing anorexia, but not causally associated with bulimia.

**Conclusion:**

Genetically predicted bulimia may have a negative association with the onset of UC, while genetically predicted anorexia does not show a causal relationship with the development of UC. Conversely, genetically predicted UC may have a negative association with the development of anorexia.

## 1 Introduction

Ulcerative colitis (UC) is a type of inflammatory bowel disease (IBD) characterized by chronic inflammation of the colon and rectum (1). The disease typically begins in the distal regions of the colon and may progress proximally to involve the entire colon. The prevalence of UC is increasing within the population (1), with its development attributed to complex interactions between environmental factors, eating disorders, the immune system, gut microbiota, and genetic predispositions (2, 3). Limited research exists on the association between eating disorders and IBD (4), primarily consisting of case reports and general studies on autoimmune disorders in individuals with eating disorders (5, 6). Those with IBD may encounter abdominal pain and bowel movement difficulties during meals, potentially leading them to selectively exclude or prefer certain foods. Consequently, dietary habits may be linked to symptoms of eating disorders in patients with UC (7). The primary eating disorders of concern are bulimia nervosa and anorexia nervosa, with research indicating a potential link between IBD and eating disorders, though the underlying mechanisms remain uncertain. Recognizing this association is crucial, as prompt identification of this comorbidity may lead to improved outcomes. One hypothesis suggests that the chronic inflammation associated with UC may impact the hypothalamic-pituitary-adrenal (HPA) axis, which plays a critical role in regulating stress and appetite (8, 9). This dysregulation could potentially lead to the development of eating disorders in UC patients. Conversely, the psychological stress and abnormal eating behaviors seen in eating disorders may exacerbate the immune response and inflammation, thereby increasing the risk of developing UC. The bidirectional risk pattern observed from the above findings suggests that eating disorders and ulcerative colitis may share a common pathogenesis and may be a third mediating variable leading to the association of these diseases.

Mendelian randomization (MR) is a methodological approach utilized in epidemiological research to investigate causal relationships between exposure factors and outcomes by leveraging genetic variants as instrumental variables with robust associations with the exposure factors (10). Due to the random assignment of germline genetic variation during meiosis, MR designs effectively reduce confounding variables and remain unaffected by environmental factors, thereby enhancing the validity of causal inference. The establishment of a validated MR association is predicated upon three fundamental assumptions: (1) the assumption of association, which posits a robust association between single nucleotide polymorphisms (SNPs) and exposure factors; (2) the assumption of independence, which asserts that SNPs are not influenced by confounders, thereby ensuring that genetic variation is not linked to confounding variables; and (3) the assumption of exclusivity, which stipulates that SNPs can solely impact the outcome through exposure factors, thereby precluding any direct effect of genetic variation on the outcome (11). Despite an exhaustive search, no MR studies examining the relationship between eating disorders (specifically anorexia nervosa and bulimia nervosa) and ulcerative colitis were identified. In order to mitigate the impact of potential confounding variables on establishing causality, the present study employs MR to explore the causal association between anorexia nervosa, bulimia nervosa, and ulcerative colitis.

## 2 Methods

### 2.1 Study design

**Figure 1:**
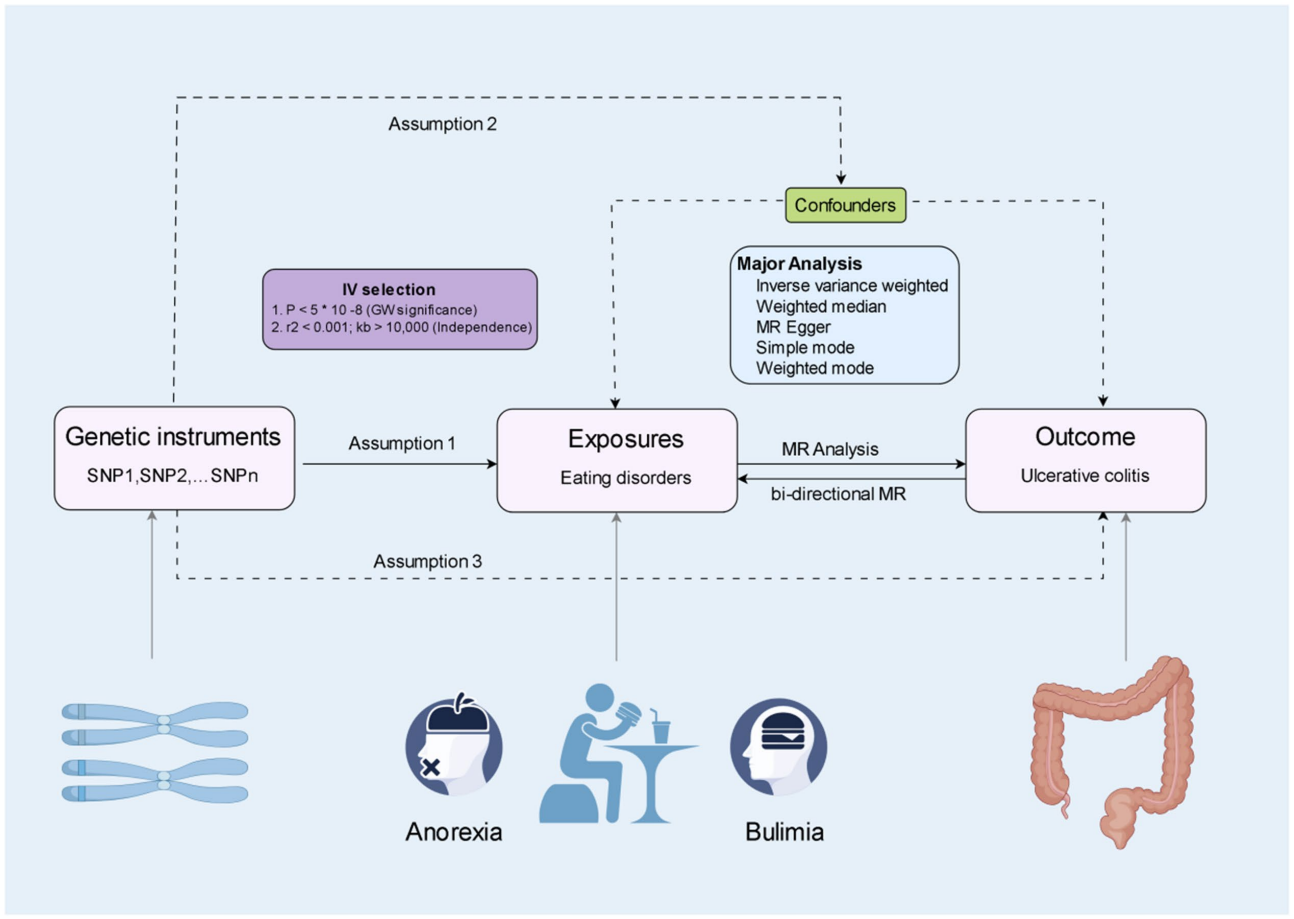
The three main assumptions of Mendelian randomisation (By Figdraw).

Figure 1 illustrate the representation of Mendelian randomization assumptions and the construction of the study design. This study utilizes publicly available data from published genome-wide association studies (GWASs) conducted by the UK Biobank (12),^1^ FinnGen study (FinnGen Documentation of R10 release https://finngen.gitbook.io/documentation/), and the International Inflammatory Bowel Disease Genetics Consortium (IIBDGC) (13). No further ethical review was deemed necessary for this MR study, as the GWAS studies incorporated in the analysis had already received approval from the relevant institutional review boards and ethics committees.

### 2.2 Instrumental variable selection

We initially identified GWASs in the FinnGen database on the two exposures of anorexia nervosa (European ethnicity, 1,039 cases and 213,826 controls; https://storage.googleapis.com/finngen-public-data-r10/summary_stats/finngen_R10_F5_ANOREX.gz) and bulimia nervosa (European ethnicity, 547 cases and 213,826 controls; https://storage.googleapis.com/finngen-public-data-r10/summary_stats/finngen_R10_F5_BULIMIA.gz) by searching databases of commonly used GWAS studies. Even though GWASs on anorexia nervosa and bulimia nervosa were conducted in UK Biobank samples in which some of SNPs were identified, we decided not to apply this option due to the small sample size (*n* = 50,068) (14).

To comprehensively assess the impact of genetic predispositions for anorexia nervosa and bulimia nervosa on the risk of UC and to obtain suitable instrumental variables (IVs), we have selected qualified genetic instruments based on the following criteria: (1) SNPs should be associated with these exposures at a genome-wide significance level (*p* < 5 × 10^−8^); for traits detected by <2 SNPs, including genome-wide significant associations (*p* < 1 × 10^−6^) or effective SNPs; (2) SNPs should be independently associated with exposures, that is, not in linkage disequilibrium with other SNPs associated with the same exposure (defined as *r*^2^ < 0.001, kb > 10,000); (3) the chosen instrumental variables should explain at least 0.1% of the variance in exposure to ensure sufficient strength of the genetic instruments for causal effect estimation (15, 16).

The genetic instruments utilized for the two exposures and the exclusion of confounding factors are outlined in Supplementary Table S1. Following the application of specified criteria and the filtering of confounding factors through the website (www.phenoscanner.medschl.cam.ac.uk, with a filtering threshold of *p*-value <0.001), 10 SNPs were found to be associated with bulimia nervosa and 5 SNPs with anorexia nervosa.

### 2.3 The acquisition of outcome data

The summary-level GWAS data for UC can be obtained from the UK Biobank (12), FinnGen study (FinnGen Documentation of R10 release https://finngen.gitbook.io/documentation/), and IIBDGC (13). The UK Biobank Study, a substantial multicenter cohort study, enrolled more than 500,000 European participants in the UK between 2006 and 2010. Summary statistics on genetic associations within the UK Biobank were derived from a Genome-Wide Association Study (GWAS) carried out in Lee’s laboratory (17). Ulcerative Colitis was identified in a study population consisting of 3,195 cases and 334,783 controls, with diagnostic criteria based on the International Classification of Diseases, 9th Revision (ICD-9) code 556.9 and 10th Revision (ICD-10) code K51. Genetic-disease associations were determined through logistic regression analysis, controlling for genetic principal components, sex, and year of birth. Summary level estimates of genetic associations for UC were also acquired in the most recent publicly accessible R10 data release of the FinnGen study, a comprehensive national cohort study launched in 2017 that integrates genetic information from the Finnish Biobank with digital health record data from the Finnish Health Registry. The UC diagnoses, consisting of 4,320 cases and 210,300 controls, were classified based on the ICD-9 (556) and ICD-10 (K51) coding systems. Genome-wide association analyses for each trait were controlled for variables including sex, age, genetic composition, and genotyping batch. The International Inflammatory Bowel Disease Genetics Consortium (IIBDGC) integrates genome-wide genotyping data and whole genome sequencing data from a cohort of more than 75,000 individuals diagnosed with IBD (13). Our analysis focuses on the European pedigree level of aggregation, utilizing data from 6,968 UC cases and 20,464 controls. The diagnosis of UC in IIBDGC was established through established radiological, endoscopic, and histopathological evaluations. Genetic associations were determined using logistic regression, controlling for age, sex, and genetic principal components.

### 2.4 Statistical analysis

The primary analyses utilized the inverse variance weighting method, along with additional methods such as MR Egger, Weighted median, Simple mode, and weighted mode (18, 19). In cases where exposures had more than 3 SNPs, the variance was aggregated and calculated using the random multiplicative effects inverse variance weighting method. For exposures containing only two SNPs, fixed-effects inverse variance weighting was employed (20, 21). The inverse variance weighting method yields the most precise estimates under the assumption that all SNPs serve as valid instruments and that pleiotropy is effectively balanced (22). In MR-Egger’s hypothesis, we consider the presence of an intercept term and use it to assess polytropy, assuming that all SNPs are invalid. If this intercept term is very close to 0, then the MR-Egger regression model is very close to IVW, but if the intercept term is very different from 0, then it suggests that there may be horizontal pleiotropy present between these IVs (23, 24). The Weighted median method, on the other hand, is a tool that assumes that more than half of the SNPs are valid (25, 26). The heterogeneity among estimates derived from individual SNPs was evaluated using Cochran’s *Q* statistic (27). In cases where the exposure was represented by only one SNP, the Wald ratio method was employed, dividing the estimate of the SNP-outcome association by the estimate of the SNP-exposure association to determine causality (28).

In order to assess the presence of bias against MR or other exposures, multiple MR methods were employed for comparisons, including leave-one-out and MR pleiotropy residual sums and outliers (MR-PRESSO) methods as sensitivity analyses (18, 29, 30). The utilization of the intercept test in MR-Egger regression may serve as a diagnostic tool for detecting horizontal multidirectionality (31). The leave-one-out method is a frequently employed technique in MR studies to evaluate the influence of individual loci on the outcomes in order to examine the robustness and validity of the model (32). MR-PRESSO method employs a global test to detect horizontal pleiotropy among SNPs, identifies outliers and pleiotropic SNPs, and yields results comparable to the IVW method in the absence of outliers (33). The *F*-statistic was utilized to assess the efficacy of the instrument for each exposure, with an *F*-statistic exceeding 10 signifying a robust instrument, while an *F*-statistic below 10 indicated a weak instrumental variable (34). Power analyses were conducted using the online web tool mRnd (15, 35).^2^ The analyses were conducted bilaterally using the TwoSampleMR (36, 37) and data.table packages (38), as well as the MR-PRESSO R package (29, 39) within the R software version 4.3.1.

## 3 Results

The study population characteristics were sourced from the UK Biobank, FinnGen Biobank, and the IIBDGC. The UK Biobank cohort primarily included individuals of European ancestry, aged 40 to 69 years, with a balanced representation of genders and comprehensive socioeconomic data, such as income, education level, and occupation. Similarly, the FinnGen Biobank focused on individuals of European descent across a broad age range, with detailed demographic and clinical information linked to national health registries. The IIBDGC dataset incorporated multiple European cohorts, ensuring diversity in age, sex, and socioeconomic status.

The determination of the sample size was based on the availability of GWAS data. For anorexia nervosa, the study utilized data from 1,039 cases and 213,826 controls of European ancestry, while for bulimia nervosa, the dataset comprised 547 cases and 213,826 controls. UC outcome data were obtained from three primary sources: the UK Biobank (3,195 UC cases and 334,783 controls), the FinnGen Biobank (4,320 UC cases and 210,300 controls), and the IIBDGC (6,968 UC cases and 20,464 controls).

**Figure 2:**
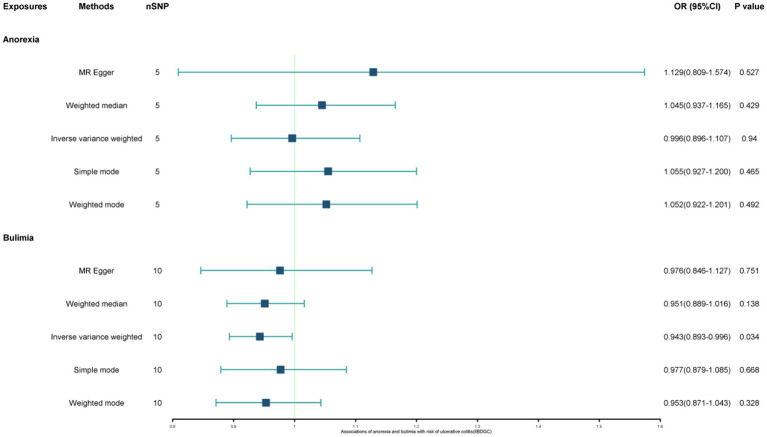
Associations of anorexia and bulimia with risk of ulcerative colitis (IIBDGC). CI, confidence interval; OR, odds ratio.

**Figure 3:**
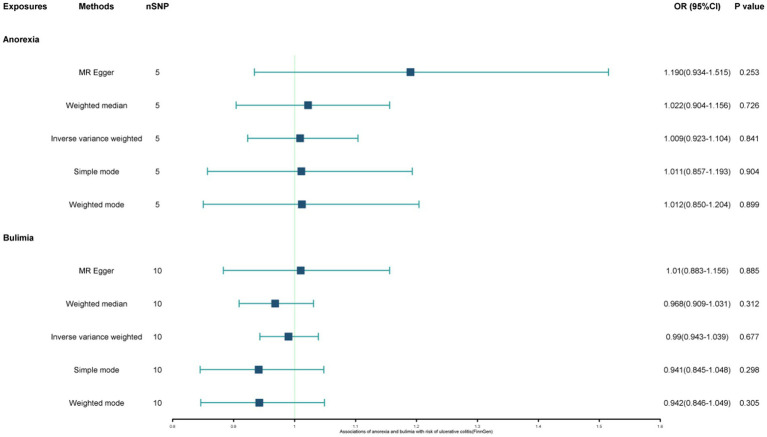
Associations of anorexia and bulimia with risk of ulcerative colitis (UK Biobank). CI, confidence interval; OR, odds ratio.

**Figure 4:**
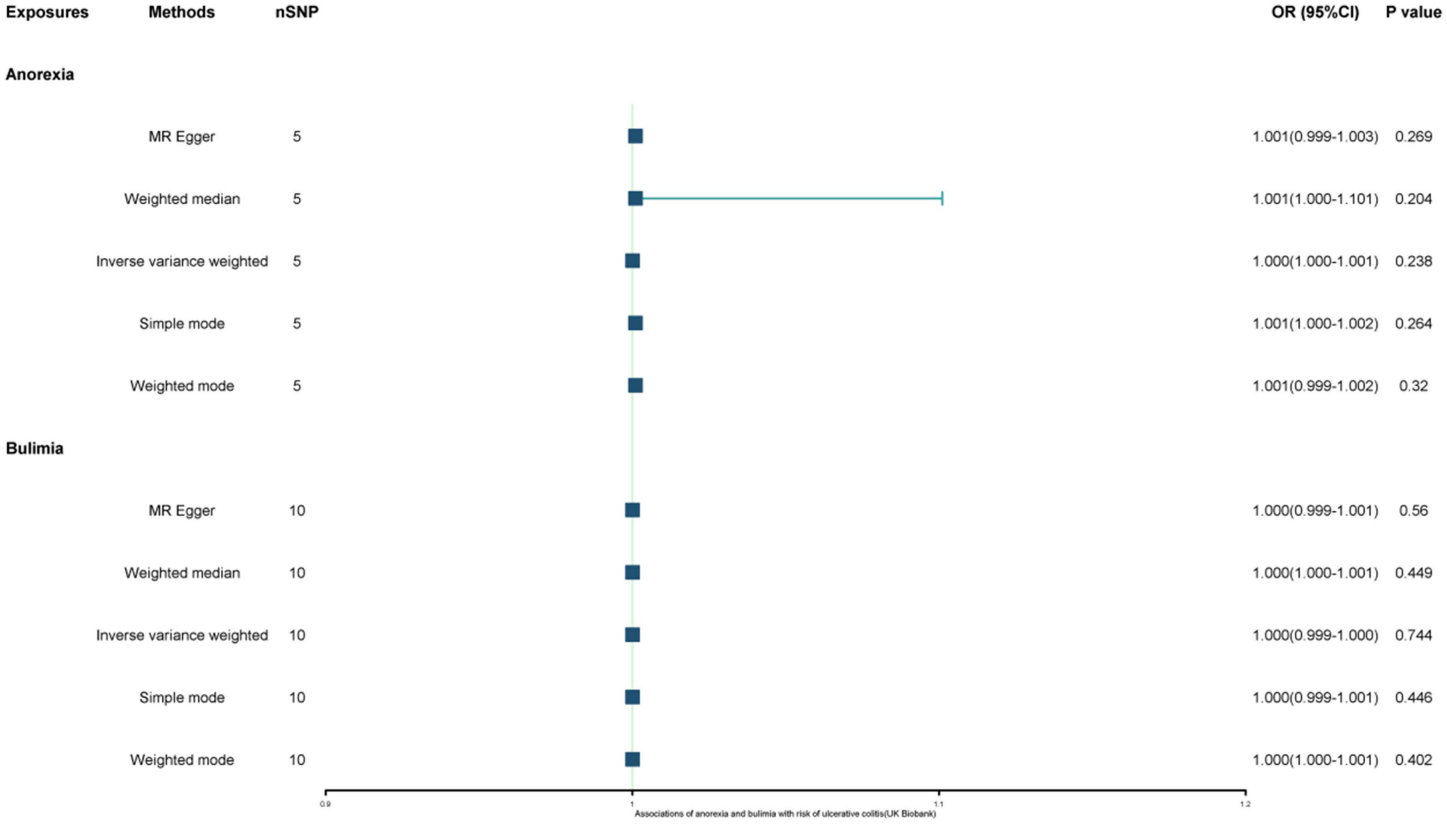
Associations of anorexia and bulimia with risk of ulcerative colitis (FinnGen study). CI, confidence interval; OR, odds ratio.

**Figure 5:**
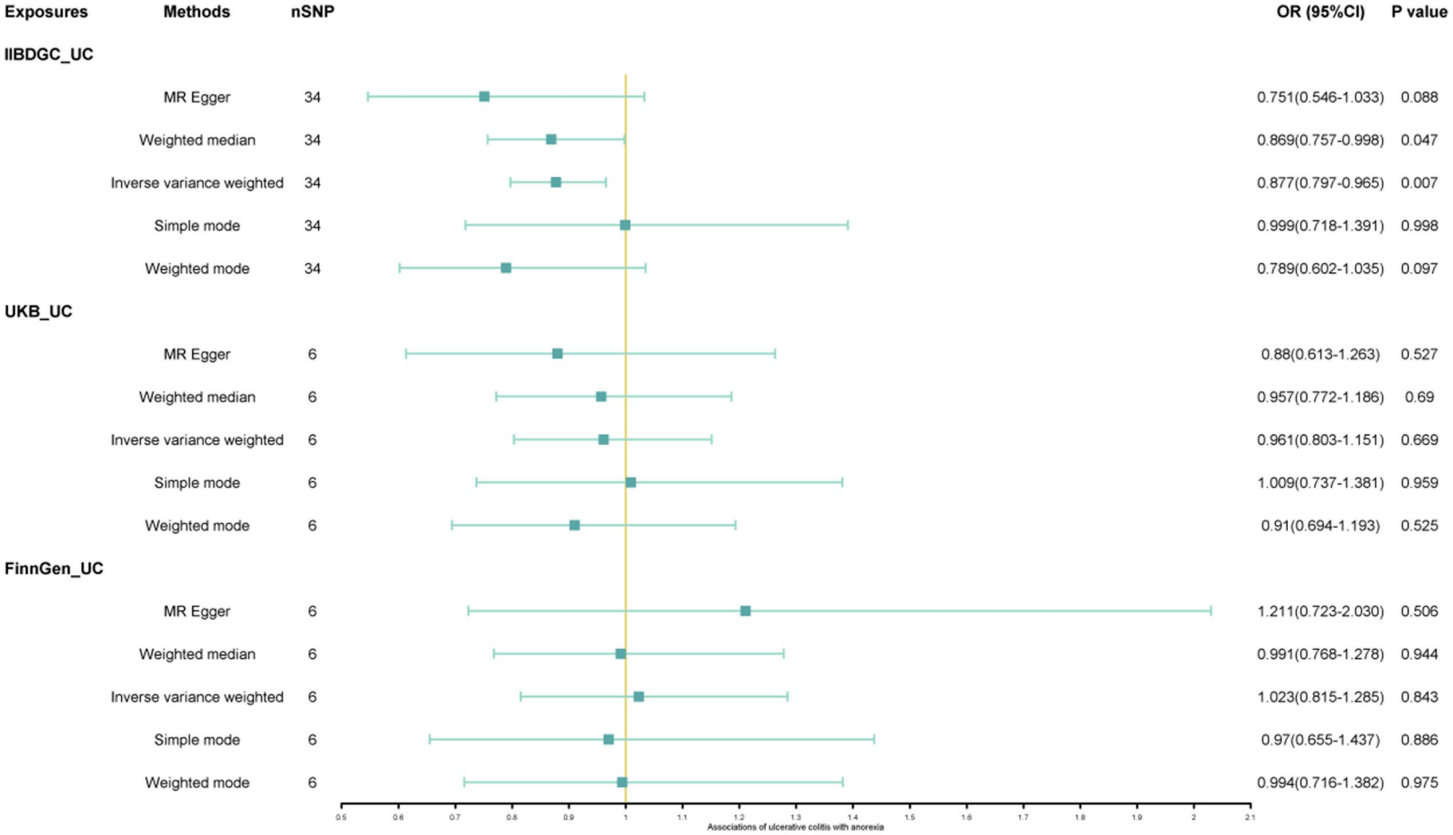
Associations of ulcerative colitis with risk of anorexia. CI, confidence interval; OR, odds ratio.

**Figure 6:**
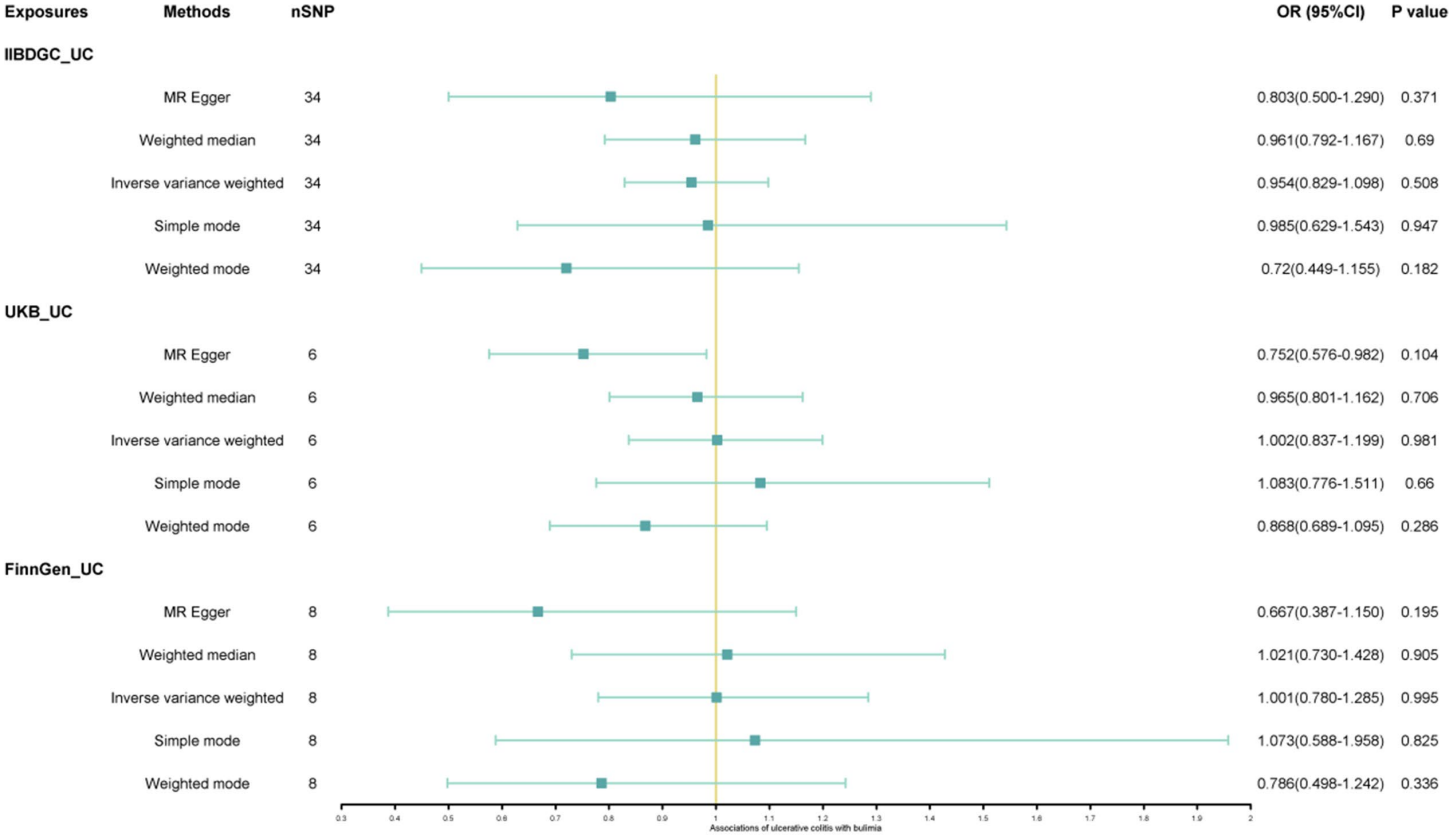
Associations of ulcerative colitis with risk of bulimia. CI, confidence interval; OR, odds ratio.

The study found a negative association between genetically predicted bulimia nervosa and the development of UC in IIBDGC (OR = 0.943, 95% CI: 0.893–0.996; *p* = 0.034). This indicates that individuals with bulimia have approximately a 5.7% lower risk of developing UC. No significant association relationship was observed in the UK Biobank (OR = 1.000, 95% CI: 0.999–1.000; *p* = 0.744) and Finnish databases (OR = 0.990, 95% CI: 0.943–1.039; *p* = 0.677). Conversely, genetically predicted anorexia was not found to be causally associated with UC, UK Biobank (OR = 1.000, 95% CI: 1.000–1.001; *p* = 0.239), Finnish databases (OR = 1.009, 95% CI: 0.923–1.104; *p* = 0.841) and IIBDGC (OR = 0.996, 95% CI: 0.896–1.107; *p* = 0.940) (Figures 2–4). In bi-directional Mendelian randomisation, UC from the IIBDGC dataset was negatively associated with the risk of anorexia (OR = 0.877, 95% CI: 0.797–0.965; *p* = 0.007), indicating that UC patients have approximately a 12.3% lower risk of developing anorexia. It is noteworthy that the weighted median method yields statistically significant results (OR = 0.869, 95% CI: 0.757–0.998; *p* = 0.047), but not causally associated with bulimia (Figures 5, 6).

The findings from the sensitivity, multiplicity, and heterogeneity analyses in the secondary investigations were largely congruent with the initial analyses, with none achieving statistical significance. This suggests that the study results were robust and dependable (Supplementary Table S2).

## 4 Discussion

In this MR analysis, we present findings suggesting a potential association between genetically predicted bulimia nervosa and a decreased risk of UC, while genetically predicted anorexia nervosa does not exhibit a significant causal relationship with UC. Additionally, our reverse MR analysis revealed a negative causal relationship between genetically predicted UC and anorexia nervosa risk, whereas no significant causal association was observed between genetically predicted bulimia nervosa and UC.

A genetic association study has determined that there is no causal relationship between anorexia nervosa and ulcerative colitis (40). A correlational study conducted in Denmark identified a causal relationship between anorexia and ulcerative colitis, while no causal association was observed between ulcerative colitis and anorexia (41). Case reports have documented an association between anorexia nervosa and the development of ulcerative colitis (42). Prior research has also demonstrated a comorbid relationship between eating disorders and IBD (4, 43). Nevertheless, a prior Mendelian randomisation study did not find evidence of a causal association, whether positive or negative, between anorexia and IBD (44). In our Mendelian randomization study, we found limited evidence to suggest a causal association between genetically predicted UC (IIBDGC) and the risk of anorexia nervosa. However, no causal relationship was observed between anorexia nervosa and UC. The disparities observed between prior observational studies, Mendelian randomization studies, and our own Mendelian randomization study may be ascribed to residual confounding, reverse causality bias, inadequate statistical power, or the more stringent inclusion criteria utilized by the IIBDGC consortium in their genome-wide association studies, which incorporate clinical presentation, gastrointestinal endoscopy, and pathology findings for diagnostic purposes. In conclusion, our research provides evidence for a potential causal link between UC and the susceptibility to anorexia nervosa, suggesting that UC may serve as a protective factor against the development of anorexia nervosa.

Bulimia nervosa and ulcerative colitis have received limited research attention, with the latter typically observed in conjunction with the former (4, 45). Our search did not yield any studies on MR examining the relationship between these two conditions. This MR study conducted by our research team has revealed a noteworthy inverse relationship between bulimia and the risk of UC. The discordant results observed may be attributed to the utilization of three databases in the present investigation, which allowed for a more accurate estimation of the association (IIBDGC). However, reverse MR analysis did not reveal a causal relationship between UC and bulimia.

The precise pathophysiological mechanisms linking eating disorders and UC are not fully understood, although several hypotheses have been proposed to elucidate this association. Specifically, attention is directed towards three key factors: (1) the influence of dietary patterns; (2) alterations in body composition resulting from pharmacological interventions for ulcerative colitis; and (3) the involvement of immuno-inflammatory processes (46). It is acknowledged that eating disorders frequently manifest as extraintestinal complications and comorbidities of UC (4). It has been suggested that dietary recommendations for patients with UC, which often include bulimic diets, are necessary to compensate for malabsorption due to disease and/or short bowel syndrome after massive bowel resection (47); in order to avoid malnutrition and/or growth retardation in young people (46). This is in line with our MR study. One hypothesis posits that elevated levels of pro-inflammatory cytokines (IL-6 and TNF-α) in patients with IBD may contribute to the perpetuation of eating disorder symptoms. These substances and hypothalamic neuropeptides exhibit similarities in their regulation of hunger/satiety and energy expenditure (48).

The European Society of Clinical Nutrition and Metabolism (ESPEN) recommends annual assessment of micronutrient levels in patients with UC, with deficiencies corrected through supplementation (49). The intersection of eating disorders and UC complicates specific recommendations for nutritional supplementation due to limited evidence. The findings of this study will supplement existing evidence from observational studies and case reports that suggest a causal link between eating disorders (specifically bulimia and anorexia) and UC, thereby enhancing the advancement of research in the area of nutritional strategies for preventing UC.

The primary strength of this study lies in its utilization of a two-sample bidirectional Mendelian randomization design, which integrates data from extensive consortia (UK Biobank, FinnGen study, and IIBDGC) to furnish robust genetic support for the identified associations. By employing data sourced from European populations, the Mendelian randomization study effectively mitigates potential confounding, reverse causation, and bias stemming from ethnic stratification. Naturally, this investigation is subject to certain limitations. The genetic tools employed in this study are capable of elucidating only a limited proportion of the variability in bulimia and anorexia, potentially resulting in the identification of modest or moderate associations. Additionally, inadequate sample sizes may also contribute to insufficient statistical power. Moreover, the limited sample sizes in genome-wide association studies (GWAS) for bulimia and anorexia may result in the erroneous selection of single nucleotide polymorphisms (SNPs). Consequently, larger GWAS are imperative to detect additional genetic variants associated with eating disorders. Additionally, a potential drawback of Mendelian randomization analyses is horizontal pleiotropy; however, no evidence of pleiotropic effects was observed in MR-Egger and PRESSO test analyses.

We hypothesized that there might be a bidirectional causal relationship between eating disorders (anorexia and bulimia) and UC. Given the limited and inconsistent evidence from previous studies, we anticipated finding some degree of association, which our study indeed confirmed for bulimia and UC in one dataset. Specifically, we expected to observe a negative association between genetically predicted eating disorders and UC, which could suggest that genetic predispositions to these conditions might influence their co-occurrence. The findings that genetically predicted bulimia is associated with a lower risk of UC, and UC with a lower risk of anorexia, were in line with our hypothesis and highlight the complexity of the genetic and environmental interactions at play. Future studies should aim to replicate these findings in larger and more diverse populations, as well as investigate the biological mechanisms underlying these associations to inform targeted prevention and treatment strategies.

## 5 Conclusion

In summary, the findings of this bidirectional Mendelian randomisation study indicate a negative association between genetically predicted bulimia and the risk of UC, specifically, individuals with bulimia have approximately a 5.7% lower risk of developing UC. While no causal relationship was observed between genetically predicted anorexia and UC. Additionally, the bi-directional Mendelian randomization analysis further revealed that UC patients have approximately a 12.3% lower risk of developing anorexia, suggesting a potential protective effect of UC against anorexia, but no significant association with bulimia. These findings provide new insights into the potential causal relationships between eating disorders and UC, highlighting the need for further research to understand the underlying mechanisms.

From an epidemiological perspective, these findings suggest that genetic predispositions to certain eating disorders may influence the risk of UC and vice versa. This underscores the importance of considering genetic factors in the prevention and management of UC and related eating disorders. Future research should focus on elucidating the underlying mechanisms driving these associations and investigating potential interventions to mitigate these risks. Understanding the genetic and biological links between these conditions could lead to more effective prevention strategies and treatments, ultimately improving patient outcomes.

## Data availability statement

The datasets presented in this study can be found in online repositories. The names of the repository/repositories and accession number(s) can be found in the article/Supplementary material.

## Ethics statement

Ethical approval was not required for the study involving humans in accordance with the local legislation and institutional requirements. Written informed consent to participate in this study was not required from the participants or the participants’ legal guardians/next of kin in accordance with the national legislation and the institutional requirements.

## Author contributions

QS: Conceptualization, Methodology, Validation, Writing – original draft. Jian Li: Formal analysis, Methodology, Validation, Writing – review & editing. YL: Data curation, Software, Validation, Writing – review & editing. MW: Data curation, Software, Validation, Writing – review & editing. Jiang Liang: Funding acquisition, Investigation, Project administration, Supervision, Visualization, Writing – review & editing. ZA: Funding acquisition, Project administration, Resources, Supervision, Writing – review & editing.

## Footnotes

^1^ http://biobank.ctsu.ox.ac.uk/crystal/field.cgi?id=K51

^2^ https://cnsgenomics.shinyapps.io/mRnd/

## References

[1] Le Berre C Honap S Peyrin-Biroulet L. Ulcerative colitis. Lancet. (2023) 402:571–84. doi: 10.1016/s0140-6736(23)00966-2 37573077

[2] Kontola K Oksanen P Huhtala H Jussila A. Increasing incidence of inflammatory bowel disease, with greatest change among the elderly: a nationwide study in Finland, 2000–2020. J Crohns Colitis. (2023) 17:706–11. doi: 10.1093/ecco-jcc/jjac177, PMID: 36420953

[3] Sazonovs A Stevens CR Venkataraman GR Yuan K Avila B Abreu MT. Large-scale sequencing identifies multiple genes and rare variants associated with Crohn’s disease susceptibility. Nat Genet. (2022) 54:1275–83. doi: 10.1038/s41588-022-01156-2, PMID: 36038634 PMC9700438

[4] Ilzarbe L Fàbrega M Quintero R Bastidas A Pintor L García-Campayo J. Inflammatory bowel disease and eating disorders: a systematized review of comorbidity. J Psychosom Res. (2017) 102:47–53. doi: 10.1016/j.jpsychores.2017.09.006, PMID: 28992897

[5] Wotton CJ James A Goldacre MJ. Coexistence of eating disorders and autoimmune diseases: record linkage cohort study, UK. Int J Eat Disord. (2016) 49:663–72. doi: 10.1002/eat.22544, PMID: 27333941

[6] Hedman A Breithaupt L Hübel C Thornton LM Tillander A Norring C. Bidirectional relationship between eating disorders and autoimmune diseases. J Child Psychol Psychiatry. (2019) 60:803–12. doi: 10.1111/jcpp.12958, PMID: 30178543

[7] Butwicka A Olén O Larsson H Halfvarson J Almqvist C Lichtenstein P. Association of childhood-onset inflammatory bowel disease with risk of psychiatric disorders and suicide attempt. JAMA Pediatr. (2019) 173:969–78. doi: 10.1001/jamapediatrics.2019.2662, PMID: 31424531 PMC6704748

[8] Fournier A Mondillon L Luminet O Canini F Mathieu N Gauchez AS. Interoceptive abilities in inflammatory bowel diseases and irritable bowel syndrome. Front Psychiatry. (2020) 11:229. doi: 10.3389/fpsyt.2020.00229, PMID: 32300314 PMC7142209

[9] Tomas C Newton J Watson S. A review of hypothalamic-pituitary-adrenal axis function in chronic fatigue syndrome. ISRN Neurosci. (2013) 2013:784520. doi: 10.1155/2013/784520, PMID: 24959566 PMC4045534

[10] Davey Smith G Hemani G. Mendelian randomization: genetic anchors for causal inference in epidemiological studies. Hum Mol Genet. (2014) 23:R89–98. doi: 10.1093/hmg/ddu328, PMID: 25064373 PMC4170722

[11] Burgess S Davey Smith G Davies NM Dudbridge F Gill D Glymour MM. Guidelines for performing Mendelian randomization investigations: update for summer 2023. Wellcome Open Res. (2019) 4:186. doi: 10.12688/wellcomeopenres.15555.3 32760811 PMC7384151

[12] Sudlow C Gallacher J Allen N Beral V Burton P Danesh J. UK biobank: an open access resource for identifying the causes of a wide range of complex diseases of middle and old age. PLoS Med. (2015) 12:e1001779. doi: 10.1371/journal.pmed.1001779, PMID: 25826379 PMC4380465

[13] Liu JZ van Sommeren S Huang H Ng SC Alberts R Takahashi A. Association analyses identify 38 susceptibility loci for inflammatory bowel disease and highlight shared genetic risk across populations. Nat Genet. (2015) 47:979–86. doi: 10.1038/ng.3359, PMID: 26192919 PMC4881818

[14] Bycroft C Freeman C Petkova D Band G Elliott LT Sharp K. The UK Biobank resource with deep phenotyping and genomic data. Nature. (2018) 562:203–9. doi: 10.1038/s41586-018-0579-z, PMID: 30305743 PMC6786975

[15] Brion MJ Shakhbazov K Visscher PM. Calculating statistical power in Mendelian randomization studies. Int J Epidemiol. (2013) 42:1497–501. doi: 10.1093/ije/dyt179, PMID: 24159078 PMC3807619

[16] Chen J Ruan X Yuan S Deng M Zhang H Sun J. Antioxidants, minerals and vitamins in relation to Crohn’s disease and ulcerative colitis: a Mendelian randomization study. Aliment Pharmacol Ther. (2023) 57:399–408. doi: 10.1111/apt.17392, PMID: 36645152 PMC11497233

[17] Zhou W Zhao Z Nielsen JB Fritsche LG LeFaive J Gagliano Taliun SA. Scalable generalized linear mixed model for region-based association tests in large biobanks and cohorts. Nat Genet. (2020) 52:634–9. doi: 10.1038/s41588-020-0621-6, PMID: 32424355 PMC7871731

[18] Burgess S Thompson SG. Interpreting findings from Mendelian randomization using the MR-Egger method. Eur J Epidemiol. (2017) 32:377–89. doi: 10.1007/s10654-017-0255-x, PMID: 28527048 PMC5506233

[19] Liu G Jin S Jiang Q. Interleukin-6 receptor and inflammatory bowel disease: a Mendelian randomization study. Gastroenterology. (2019) 156:823–4. doi: 10.1053/j.gastro.2018.09.059, PMID: 30445015

[20] Larsson SC Butterworth AS Burgess S. Mendelian randomization for cardiovascular diseases: principles and applications. Eur Heart J. (2023) 44:4913–24. doi: 10.1093/eurheartj/ehad736, PMID: 37935836 PMC10719501

[21] Long Y Tang L Zhou Y Zhao S Zhu H. Causal relationship between gut microbiota and cancers: a two-sample Mendelian randomisation study. BMC Med. (2023) 21:66. doi: 10.1186/s12916-023-02761-6, PMID: 36810112 PMC9945666

[22] Yavorska OO Burgess S. MendelianRandomization: an R package for performing Mendelian randomization analyses using summarized data. Int J Epidemiol. (2017) 46:1734–9. doi: 10.1093/ije/dyx034, PMID: 28398548 PMC5510723

[23] Ren Z Simons P Wesselius A Stehouwer CDA Brouwers M. Relationship between NAFLD and coronary artery disease: a Mendelian randomization study. Hepatology. (2023) 77:230–8. doi: 10.1002/hep.32534, PMID: 35441719 PMC9970021

[24] Chen M Xie CR Shi YZ Tang TC Zheng H. Gut microbiota and major depressive disorder: a bidirectional Mendelian randomization. J Affect Disord. (2022) 316:187–93. doi: 10.1016/j.jad.2022.08.012, PMID: 35961601

[25] Wu F Huang Y Hu J Shao Z. Mendelian randomization study of inflammatory bowel disease and bone mineral density. BMC Med. (2020) 18:312. doi: 10.1186/s12916-020-01778-5, PMID: 33167994 PMC7654011

[26] Xu J Zhang S Tian Y Si H Zeng Y Wu Y. Genetic causal association between iron status and osteoarthritis: a two-sample Mendelian randomization. Nutrients. (2022) 14:3683. doi: 10.3390/nu14183683, PMID: 36145059 PMC9501024

[27] Li P Wang H Guo L Gou X Chen G Lin D. Association between gut microbiota and preeclampsia-eclampsia: a two-sample Mendelian randomization study. BMC Med. (2022) 20:443. doi: 10.1186/s12916-022-02657-x, PMID: 36380372 PMC9667679

[28] Tasnim S Wilson SG Walsh JP Nyholt DR. Cross-trait genetic analyses indicate pleiotropy and complex causal relationships between headache and thyroid function traits. Genes. (2022) 14:16. doi: 10.3390/genes14010016, PMID: 36672757 PMC9858525

[29] Verbanck M Chen CY Neale B Do R. Detection of widespread horizontal pleiotropy in causal relationships inferred from Mendelian randomization between complex traits and diseases. Nat Genet. (2018) 50:693–8. doi: 10.1038/s41588-018-0099-7, PMID: 29686387 PMC6083837

[30] Flatby HM Ravi A Damås JK Solligård E Rogne T. Circulating levels of micronutrients and risk of infections: a Mendelian randomization study. BMC Med. (2023) 21:84. doi: 10.1186/s12916-023-02780-3, PMID: 36882828 PMC9993583

[31] Huang D Lin S He J Wang Q Zhan Y. Association between COVID-19 and telomere length: a bidirectional Mendelian randomization study. J Med Virol. (2022) 94:5345–53. doi: 10.1002/jmv.28008, PMID: 35854470 PMC9349767

[32] Xiao G He Q Liu L Zhang T Zhou M Li X. Causality of genetically determined metabolites on anxiety disorders: a two-sample Mendelian randomization study. J Transl Med. (2022) 20:475. doi: 10.1186/s12967-022-03691-2, PMID: 36266699 PMC9583573

[33] Xiang M Wang Y Gao Z Wang J Chen Q Sun Z. Exploring causal correlations between inflammatory cytokines and systemic lupus erythematosus: a Mendelian randomization. Front Immunol. (2022) 13:985729. doi: 10.3389/fimmu.2022.985729, PMID: 36741410 PMC9893779

[34] Bottigliengo D Foco L Seibler P Klein C König IR Del Greco MF. A Mendelian randomization study investigating the causal role of inflammation on Parkinson’s disease. Brain. (2022) 145:3444–53. doi: 10.1093/brain/awac193, PMID: 35656776 PMC9586538

[35] Zhao B Wang Z Liu D Zhang S. Genetically predicted serum testosterone and risk of gynecological disorders: a Mendelian randomization study. Front Endocrinol. (2023) 14:1161356. doi: 10.3389/fendo.2023.1161356, PMID: 38075074 PMC10710168

[36] Hemani G Zheng J Elsworth B Wade KH Haberland V Baird D. The MR-base platform supports systematic causal inference across the human phenome. eLife. (2018) 7:e34408. doi: 10.7554/eLife.34408, PMID: 29846171 PMC5976434

[37] Wu Y Li Y Zhu J Long J. Shared genetics and causality underlying epilepsy and attention-deficit hyperactivity disorder. Psychiatry Res. (2022) 316:114794. doi: 10.1016/j.psychres.2022.114794, PMID: 35994864

[38] van Galen P Hovestadt V Wadsworth Ii MH Hughes TK Griffin GK Battaglia S. Single-cell RNA-Seq reveals AML hierarchies relevant to disease progression and immunity. Cell. (2019) 176:1265–1281.e24. doi: 10.1016/j.cell.2019.01.031, PMID: 30827681 PMC6515904

[39] Lyu F Wang L Jia Y Wang Y Qi H Dai Z. Analysis of zinc and stromal immunity in disuse osteoporosis: Mendelian randomization and transcriptomic analysis. Orthop Surg. (2023) 15:2947–59. doi: 10.1111/os.13840, PMID: 37752822 PMC10622276

[40] Tylee DS Sun J Hess JL Tahir MA Sharma E Malik R. Genetic correlations among psychiatric and immune-related phenotypes based on genome-wide association data. Am J Med Genet B Neuropsychiatr Genet. (2018) 177:641–57. doi: 10.1002/ajmg.b.32652, PMID: 30325587 PMC6230304

[41] Larsen JT Yilmaz Z Vilhjálmsson BJ Thornton LM Benros ME Musliner KL. Anorexia nervosa and inflammatory bowel diseases-diagnostic and genetic associations. JCPP Adv. (2021) 1:e12036. doi: 10.1002/jcv2.12036, PMID: 37431410 PMC10242845

[42] Ratih SD Makrufardi F Azizah AFN Damayanti W. Multiple mesenteric lymphadenopathies in pediatric with ulcerative colitis: a case report. Radiol Case Rep. (2024) 19:600–3. doi: 10.1016/j.radcr.2023.10.046, PMID: 38074434 PMC10709117

[43] Hoffmann JC. Crohn’s disease and ulcerative colitis: in young women often misdiagnosed as anorexia. MMW Fortschr Med. (2010) 152:34. doi: 10.1007/BF03366836 PMID: 20848986

[44] Gong W Guo P Li Y Liu L Yan R Liu S. Role of the gut-brain axis in the shared genetic etiology between gastrointestinal tract diseases and psychiatric disorders: a genome-wide pleiotropic analysis. JAMA Psychiatry. (2023) 80:360–70. doi: 10.1001/jamapsychiatry.2022.4974, PMID: 36753304 PMC9909581

[45] Kuźnicki P Neubauer K. Emerging comorbidities in inflammatory bowel disease: eating disorders, alcohol and narcotics misuse. J Clin Med. (2021) 10:4623. doi: 10.3390/jcm10194623, PMID: 34640641 PMC8509435

[46] Quick VM Byrd-Bredbenner C Neumark-Sztainer D. Chronic illness and disordered eating: a discussion of the literature. Adv Nutr. (2013) 4:277–86. doi: 10.3945/an.112.003608, PMID: 23674793 PMC3650496

[47] Culkin A Gabe SM Peake ST Stern JM. A dangerous combination of binge and purge. Int J Eat Disord. (2012) 45:302–4. doi: 10.1002/eat.20912, PMID: 21433049

[48] Solmi M Santonastaso P Caccaro R Favaro A. A case of anorexia nervosa with comorbid Crohn’s disease: beneficial effects of anti-TNF-α therapy? Int J Eat Disord. (2013) 46:639–41. doi: 10.1002/eat.22153, PMID: 23813727

[49] Bischoff SC Bager P Escher J Forbes A Hébuterne X Hvas CL. ESPEN guideline on clinical nutrition in inflammatory bowel disease. Clin Nutr. (2023) 42:352–79. doi: 10.1016/j.clnu.2022.12.004, PMID: 36739756

